# On the Treatment of Poisoning by the Bite of a Rattlesnake

**Published:** 1848-09

**Authors:** Josiah Trowbridge

**Affiliations:** Buffalo


### BUFFALO MEDICAL JOURNAL

AND

### MONTHLY REVIEW.

VOL. 4. SEPTEMBER, 1848. NO. 4.

### ORIGINAL COMMUNICATIONS.

ART. I.—*On the Treatment of Poisoning by the Bite of a Rattlesnake.* By JOSIAH TROWBRIDGE, M. D.

SIR:—In your Journal for the month of July, I notice an account of the symptoms of, and treatment for the bite of a rattlesnake, in the case of the lamented Dr. Wainright, of New York, taken from the Annalist. I am not disposed to make any strictures on the treatment in that case. The bite of a rattlesnake is of rare occurrence in the Northern States of late years, as they have been nearly, if not entirely, exterminated from the settled portions of the country. I will simply relate what has occurred in my own practice.

I came to Buffalo to reside in 1811, and at that time the rattlesnake was common in many localities in the vicinity. I think some six or eight cases have fallen under my care. I distinctly recollect four of them. I recollect also, the prominent symptoms in two of the cases. The first occurred in 1812. A boy of about twelve years of age was bitten on the side of one foot, near the small toe. Being absent from the village, I did not see him until two or three hours after the accident. The limb was then badly swollen to the body, the skin was discolored and mottled, and, if I recollect aright, of a green and yellow color. The pain was severe, and the pulse accelerated. I immediately directed the administration of Olive Oil, both externally and internally: Internally, by the administration of ℥ii every half hour, and externally, constantly, with friction. Under this treatment the swelling subsided, the pain was assuaged, and the boy recovered.

The other was a man of fifty years of age, of intemperate habits. He kept two large rattlesnakes in a box. At about 10 o'clock, P. M., being somewhat intoxicated, he removed the snakes from the box to the floor, and began playing with them. At length one of them struck him between the thumb and forefinger. My office being in the vicinity, a student of mine was requested to attend. He applied salt and vinegar to the hand, and administered aqua ammoniæ internally, without relief. At half-past 11 or 12 P. M., I was called. At this time the hand and arm were badly swollen to the elbow, and the pain severe. I directed the administration of the Oil as in the former case. I requested the student to remain with him, and to see that the remedy was faithfully applied until the threatening symptoms subsided. The next morning, at 7 or 8 o'clock, I met the patient upon the side-walk, free from any ill effect from the poison. All the other cases were treated in the same way, and all recovered.

I am aware that numerous trials and long experience are required to establish the character and value of any remedy, and I have not great confidence in antidotes, and less in specifics. In looking back into some of the old medical periodicals, I find that Sweet Oil, as an antidote to the poison of the rattlesnake, is not a new remedy. In Vol. 2d, No. 2, Article 4th, of the Medical Repository, published in 1805, there is an article republished from the Charleston (South Carolina) City Gazette, a part of which I quote. "In great cities, particularly in London, a number of persons procure their livelihood by catching vipers. They are employed by Chemists and Apothecaries, &c. I remember some years before leaving England, to have read in the Philosophical Transactions of the Royal Society in London, a curious circumstance relative to one of these viper catchers. A member of the Society had received, causually, imformation that a man engaged in this business was frequently bitten, and that he cured himself with Olive Oil. After considerable inquiry the viper catcher was found, and the questions asked, whether he did cure himself with the Oil, and whether he was willing to gratify a number of gentlemen by an exhibition of the fact? The man answered affirmatively to both questions. Accordingly a most numerous meeting of the Royal Society was convened, composed of a considerable number of the nobility, &c. The viper catcher attended, accompanied by his wife, with a large viper, and laying his arm naked to the shoulder, suffered the irritated reptile to strike, which it did forcibly. His wife permitted the poison to operate till her husband's head, face and tongue were greatly swelled, his arm and face also very black, and his senses much affected, when she applied the oil, by pouring a small quantity down, and bathing the part bitten. The man gradually, and soon recovered. This circumstance being strongly impressed upon my mind, and knowing that the poison of an English viper is considered, in that country, the most subtle in nature, determined me to try its antidotal power in the bite of the rattlesnake, the first opportunity which should offer, on my retirement from Charleston to the back Country, now Pendleton County. I was also particularly impelled to make the trial from a consideration of the newness and wildness of the country, and the number of my family, beside which, there were hardly a dozen more in the county. This was in the year 1786. In about a month after my arrival, a person in full speed came to my camp, and most urgently begged to know if I could assist a man who had just been bitten by a large rattlesnake. Although I lamented the misfortune, I rejoiced at the opportunity it offered to ascertain fully the property of Olive Oil as an antidote to this deadly poison. Accordingly I put a vial of oil in my pocket, and mounted the messenger's horse. When I arrived at the unfortunate man's cabin, he struck me as the most frightful object I had ever beheld. His hands and face were prodigiously swelled, the latter black, his tongue proportionably enlarged and out of his mouth, his eyes as if shooting from their sockets, his senses gone, and every appearance of immediate suffocation. He had been struck on the side of the foot, about the middle, in the hollow. Immediately, but with great difficulty, I got down two table-spoonfulls of oil. Its effects were almost instantaneous, and astonishingly powerful, in counteracting the poison, as appeared by the strong, though quick convulsions that followed. In about thirty minutes it operated strongly, both emetically and cathartically, after which the swelling of the head and face &c. gradually abated, and the tongue began to assume its place. In about two hours, he was so far recovered as to be able to articulate, and from that time recovered fast. The oil inwardly taken, and applied to the foot and leg, both exceedingly swelled, did not exceed seven or eight spoonfulls.

The number of cases of a like nature, in the course of twelve years, has been considerable, in all of which Olive Oil has proved itself to be peculiarly adapted, and fully adequate to the worst of cases, if timely applied."

I find another case recorded in the eighth volume of the Medical Journal of Medical Sciences, page 397, communicated by Dr. Horner, which terminated fatally. It appears that ℥iiss of Olive Oil was administered in two doses, at a long interval between, and the last dose when the man was in *articulo mortis*. The principal reliance in this case seems to have been upon cupping, external irritants, aqua ammoniæ, &c. I do not think that this case makes either for, or against the use of Olive Oil. I copy from the Boston Medical and Surgical Jonrnal of January 5th, 1848, from an article communicated by Dr. Stephen W. Williams, of Deerfield, Massachusetts, the following additional testimony in favor of the use of Olive Oil. "The use of Olive Oil may be strongly recommended for the bite of the rattlesnake. My grandfather, Dr. Thos. Williams, formerly a very distinguished physician in this town, once restored a patient bitten by a rattlesnake, apparently in the last stage of life, by the external and internal administration of Olive Oil. Mr. J. Miller, of Pendleton, of South Carolina, observes, that Olive Oil, taken internally in the quantity of a few spoonfulls, and applied to the bitten part, has proved itself fully adequate to the worst cases if timely administered."

It seems to me that the results in the cases here related, furnish abundant testimony in favor of an early and diligent use of this remedy. That there may be cases which will be fatal under any circumstances, and in spite of the Olive Oil, or any other remedy, is probable, but if it succeeds in a large majority, it is sufficient to warrant its use, and a reasonable reliance upon its powers as an antidote to the poison.

Buffalo, August, 1848.

